# Analysis of Fuel Alternative Products Obtained by the Pyrolysis of Diverse Types of Plastic Materials Isolated from a Dumpsite Origin in Pakistan

**DOI:** 10.3390/polym15010024

**Published:** 2022-12-21

**Authors:** Nuzhat Javed, Sana Muhammad, Shazia Iram, Muhammad Wajahat Ramay, Shaan Bibi Jaffri, Mariem Damak, György Fekete, Zsolt Varga, András Székács, László Aleksza

**Affiliations:** 1 Department of Environmental Sciences, Fatima Jinnah Women University, The Mall, Rawalpindi 46000, Pakistan; 2 Institute of Environmental Sciences, Hungarian University of Agriculture and Life Sciences, Páter Károly u. 1, H-2100 Gödöllő, Hungary; 3 Profikomp Environmental Technologies Inc., Kühne Ede u. 7, H-2100 Gödöllő, Hungary

**Keywords:** pyrolysis, hydrocarbons, FT-IR, GC-MS, condensation, fuel

## Abstract

The current energy crisis and waste management problems have compelled people to find alternatives to conventional non-renewable fuels and utilize waste to recover energy. Pyrolysis of plastics, which make up a considerable portion of municipal and industrial waste, has emerged as a feasible resolution to both satisfy our energy needs and mitigate the issue of plastic waste. This study was therefore conducted to find a solution for plastic waste management problems, as well as to find an alternative to mitigate the current energy crisis. Pyrolysis of five of the most commonly used plastics, polyethylene terephthalate (PET), high- and low-density polyethylene (HDPE, LDPE), polypropylene (PP), and polystyrene (PS), was executed in a pyrolytic reactor designed utilizing a cylindrical shaped stainless steel container with pressure and temperature gauges and a condenser to cool down the hydrocarbons produced. The liquid products collected were highly flammable and their chemical properties revealed them as fuel alternatives. Among them, the highest yield of fuel conversion (82%) was observed for HDPE followed by PP, PS, LDPE, PS, and PET (61.8%, 58.0%, 50.0%, and 11.0%, respectively). The calorific values of the products, 46.2, 46.2, 45.9, 42.8 and 42.4 MJ/kg for LPDE, PP, HPDE, PS, and PET, respectively, were comparable to those of diesel and gasoline. Spectroscopic and chromatographic analysis proved the presence of alkanes and alkenes with carbon number ranges of C_9_–C_15_, C_9_–C_24_, C_10_–C_21_, C_10_–C_28_, and C_9_–C_17_ for PP, PET, HDPE, LDPE, and PS, respectively. If implemented, the study will prove to be beneficial and contribute to mitigating the major energy and environmental issues of developing countries, as well as enhance entrepreneurship opportunities by replicating the process at small-scale and industrial levels.

## 1. Introduction

Plastic waste is contemplated as one of the world’s major environmental problems. Plastic products are used all over the world, resulting in immense quantities of persistent waste and pollution of land and oceans [1]. The three main sources of plastic wastes are technological waste (from production and processing), industrial waste (from processing and confectioning), and waste from worn-out plastic products (packaging materials and worn-out products) [2]. Plastic production and corresponding waste generation has approximately doubled every twenty years [3].It is estimated Pakistan alone generated more than two million metric tons of plastic waste in 2016, whereas the US generated the greatest amount of plastic waste at 42 million metric tons [4], causing hazards to marine life and ecosystems [5]. The main environmental impacts and concerns related to plastic waste include: (a) decomposition times (half-lives) can be extremely long, even centuries under isolated circumstances; (b) fine particles (microplastics and nanoplastics can be formed via dispersion processes); and (c) toxicity (reprotoxic, carcinogenic, or hormonally active substances can be generated from plastics or their additives during production and extended exposure [2]. This calls for urgent attention to prevent, reduce, reuse, and recycle plastic wastes and the development of recovery methods to generate energy [2,6,7]. Nonetheless, reused plastic will end its lifecycle in landfills or oceans [7]; therefore, recycling or utilizing plastic waste is a more beneficial option [8,9]. Developing countries in South Asia, such as Pakistan, do not have a formal waste management system, which leads to the dumping of plastics unattended. This further escalates the existing problem of waste management in these countries [10]. Unfortunately, due to the country’s lack of effective enforcement of environmental protection laws against waste dumping, degradable waste is dumped unsegregated alongside non-biodegradable plastic waste on land and in rivers [11]. Various solutions have been suggested to mitigate this issue, such as recycling and use of bioplastics [12,13], among which biogas generation has also been proposed [14]. However, these solutions require structural changes that are harder to implement in developing economies where sustainability is often difficult to sufficiently take into consideration. Moreover, the energy and electricity shortage in Pakistan has crippled the economy [15]. Due to the current global energy crisis, developing countries such as Pakistan have suffered immensely due to rising fuel prices and energy deficits [16]. Pakistan is already under energy stress as the conventional fuel resources are non-renewable and there is no alternative energy production system that is both sustainable and efficient [17]. To meet the energy needs of the rapidly growing population, quick and sustainable solutions are needed to address the energy crisis.

In order to overcome the problem of plastic waste management, along with the energy shortfall, a possible resolution is energy recovery from plastic wastes as synthetic petrochemical-based products [18], resulting not only in energy retrieval but also significant reductions in the volume of plastic waste [19]. Nearly 6% of the petroleum products used globally are used in the manufacturing of plastics [20]. The best method for turning plastic waste into fuel is thermal deterioration, such as pyrolysis [21,22]. The technology of conversion of various plastics into fuel (often termed waste plastic fuel or WPF) is now considered an approach to achieving a circular economy [23,24,25,26,27]. The technology of thermal conversion of plastics has been advocated worldwide [21,28,29,30,31,32], and research is advancing from the use of clean plastic materials to mixed plastic starting materials [26,27,33,34], even plastic recovery from landfills [22]. Nonetheless, the use of blended plastics presents tremendous technological difficulties, e.g., adverse nitrogen substances formed during pyrolysis [24]. Various technologies are being developed, such as using additives [27], catalysis [33,35,36,37,38], removal of N-compounds formed during pyrolysis prior to further refining [24], or the use of plastic waste and desiccated sludge together [34], but efforts continue to focus on individual plastic substances. Antagonists of plastic-to-fuel (PTF) technologies dispute that that they are energy-demanding, exacerbate combustion gas emissions, and perpetuate the overproduction of plastic [28,38]. Indeed, it has to be admitted that these pyrolysis technologies, with or without catalysis, should certainly not be used to advocate plastic production, but they offer utility in favorably eliminating plastic waste [28] and also reducing municipal solid waste (MSW). In fact, conversion of clean plastic materials to fuel products has not only been proposed as a feasible technology, but it is also a subject of economic analysis [39].

As the world attempts to cope with the major energy crisis, the use of renewable sources of energy is being advocated globally more than ever. Within the objectives of the European Green Deal [38], particular emphasis has been placed on the use of renewable energy resources in the European Union to achieve 50–55% reduction in the emission of greenhouse gases by 2030 and reach climate neutrality by 2050 on the one hand, while also achieving 65% waste recycling by 2035 and eliminating waste disposal on the other hand. In Hungary, a new Centre for Circular Economy Analysis and Knowledge has been established at the Hungarian University of Agriculture and Life Sciences [39], which fosters dissemination of the most recent knowledge on the circular economy, its implementation at domestic and international levels, as well as the assessment and on-site utilization of biodegradable waste generated at the campus site. Based on experience gathered in Hungary [40], operational models and management advice is also provided for international collaborations. With the data gathered from studies in developing countries such as Pakistan, a possible resolution could be derived for the energy and waste management problems and also encourage sustainable development in countries worldwide through alternative methods of energy recovery.

Therefore, in order to simultaneously address the issues of energy shortage and plastic waste, pyrolysis of polymers to recover energy in the form of fuel or gas has emerged as a frontrunner in recent years [41]. Studies have been conducted to analyze the products of pyrolysis from either individual or waste mixtures [30,31,32,33,34]. A study by Quesada et al. [42] found that the pyrolysis of commonly used plastics (PE, PS, and PP) and their binary and tertiary mixtures generated products that possessed characteristics similar to conventional fossil fuels, such as diesel. The products exhibited high calorific values and compositions which were comparable to commercial fuels. Miandad et al. [43] reported that the heating values of fuels obtained from the pyrolysis of plastics, such as LDPE, PET, and HDPE, were comparable to those of diesel and gasoline, which reflected their feasibility as potential alternative energy sources. While reviewing pyrolysis products, Mangesh et al. [44] suggested that due to the immiscibility of these crude fuels with conventional fuels, further processing and refining would be required by oil refineries to obtain high quality gasoline and diesel. Sogancioglu et al. [45] found C_10_–C_40_ hydrocarbons in HDPE-derived fuel, whereas most studies revealed an increase in gaseous products with increasing temperature and a decrease in liquid yield [43]. Singh et al. [30] used a blend of 50% pyrolysis oil from mixed plastic waste and diesel in an ignition engine and found the quality of the fuel comparable to conventional fuels, with high brake thermal efficiency and lower fuel consumption. However, this blend produced more carbon monoxide emissions. A blend of 5% pyrolysis fuel and diesel was found to be comparable to the standard for diesel, which implied its practicability in engines [46], but further distillation and refining was required to obtain better results. These studies indicated the potential of pyrolysis products to be used as substitutes for conventional non-renewable fuels, but the authors did not regard cost-effectiveness and feedstock significance, which are important factors to ensure the commercialization of these products at small and large scales, as pointed out by Bai and Givens [47], Jahirul et al. [32], Al-Salem et al. [48], and Thewys and Kuppens [49]. Jahirul et al. [32] also indicated that cost-effectiveness is important to attain positive results and promote this technology. Furthermore, the conditions of developing countries are dissimilar; formal waste management and segregation are nonexistent in these countries [32,47]. The findings of this study are novel as they incorporate the conditions of a developing country, such as Pakistan, and demonstrate the pyrolysis of the most commonly used plastics from a dumpsite origin in a cost-effective and sustainable manner. This is important for countries such as Pakistan and points out the replicability of the process at smaller scales, which will enhance entrepreneurship opportunities, the economy, and address problems of waste management and the energy crisis. The results reflect the possibility of using pyrolysis fuel as an alternative for conventional fuels at a larger scale, but can also be used for ignition in domestic circles at a smaller scale.

The objective of this study was to provide an efficient solution to mitigate the issues of energy crisis and plastic waste management by successful pyrolysis of the most abundantly found plastics in MSW, i.e., PET, LDPE, HDPE, PP, and PS, using a cost-effective method and analyze the physio-chemical characteristics of the products. Plastic waste is assessed is a potential resource for fuel alternatives and provides a mean to reduce the volume of plastic waste in landfills by recovering energy in the form of crude fuel. The pyrolysis products showed high yields, calorific values, and chemical properties comparable to those of conventional fuels, denoting that if replicated, the technology will generate entrepreneurship opportunities, curtail the issues of energy shortage and plastic waste, and contribute to sustainable development.

## 2. Materials and Methods

### 2.1. Types of Plastics

Five types of plastic materials (Table 1), including polyethylene terephthalate (PET), high-density polyethylene (HDPE), low-density polyethylene (LDPE), polypropylene (PP), and polystyrene (PS) were used to perform the experiments. These five plastics were selected because they make up the majority of the volume of plastic waste, (including MSW) since these plastics are the most commonly used domestically [45,50,51,52,53]. They can be easily segregated from MSW (see Supplementary Materials Figure S1). The selection of various polymers enabled a comparison of the quality of each derived fuel. All of the plastic samples were gathered from waste locations and transported to the lab where the experiments were conducted. They were washed with tap water and then subjected to thermal decomposition in a pyrolysis reactor.

### 2.2. Design of the Pyrolysis Reactor and Conversion of Plastics into Fuels of Different Quality

**Figure 1:**
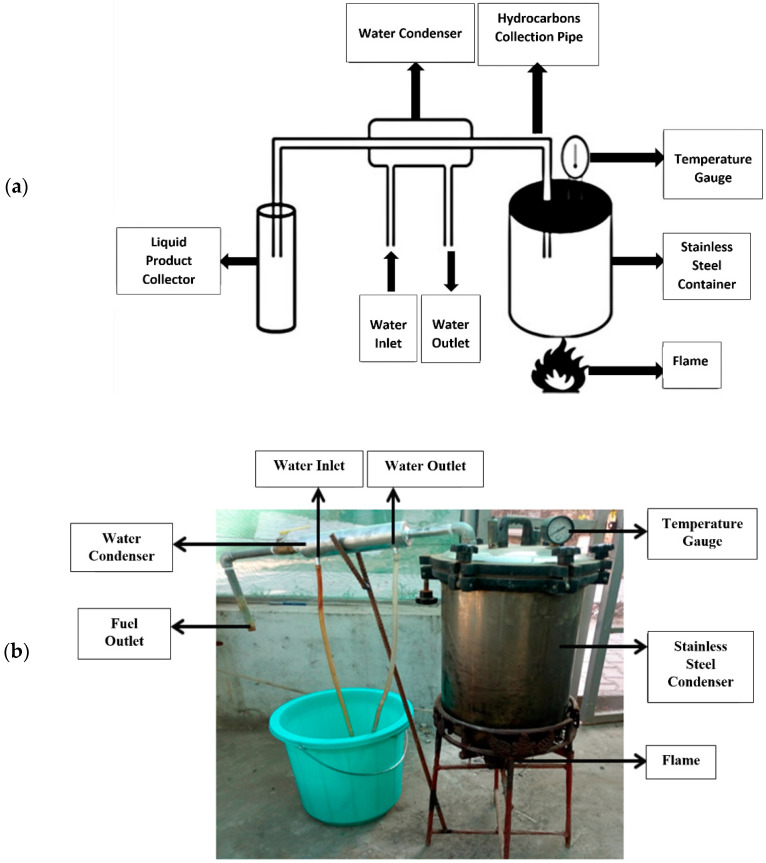
The pyrolysis set-up used in the study. (**a**) Schematic representation. (**b**) A photograph of the equipment for the conversion of plastics into fuel.

After a preliminary pyrolysis pilot study using glass equipment (see Supplementary Materials Figure S2), pyrolysis was carried out in a steel reactor. Figure 1 depicts the pyrolysis reactor utilized in the investigation, which corresponded to the design used by Oni and Ayodeji [54] and Kumar and Singh [55] in their studies. It encompasses a condensation unit and a stainless-steel container. A temperature gauge was installed on the container’s lid. When the lid was closed, an inert atmosphere was created, and air could not interfere with the chemical reaction occurring inside the reactor because the container was maintained with airtight insulation. Maintenance of pressure and condensation were key elements to ensure that off gases could escape, and an inert atmosphere was maintained inside the reactor. Condensation facilitated separation of the steam. The condensate was collected using a water condenser. Iron metal made up the condenser, which included a water intake and outlet to create a concurrent cooling water flow from a bucket used as a reservoir and a water pump used to keep the water flowing. To reduce the amount of hydrocarbons lost, and increase the yield of fuel, ice was used for condensation purposes. A pyrolysis temperature of 450 °C was maintained for PS and HDPE, whereas 500–550 °C was maintained for LDPE, PP, and PET. These temperatures were comparable to those used in previous studies [43,47,50,56]. Constant observations of pressure and flame were performed to ensure maximum yield. Higher temperatures are attributed to the production of gases, whereas low temperatures are attributed to the production of liquid product [56]. To thermally degrade the plastic in the container, flame heating was used. The melted plastic released hydrocarbon vapors, which condensed into liquids and were collected in a glass bottle at the end of the cooling pipe (see Supplementary Materials Figures S3 and S4). The average time taken by the reactor to convert plastics into fuel was approximately 90 min.

### 2.3. Analysis of the Fuel Obtained by Pyrolysis of Plastics

Categorization of the obtained fuels was conducted based on their physical and chemical properties. Physical analysis of the fuel included yield, color, density, viscosity, calorific value, by-products, and rate of conversion of plastic into other products.

#### 2.3.1. Yield, Color, Density, and Viscosity

To determine the yield of the obtained fuel, the mass of the derived fuel from each type of plastic was measured separately, divided by the mass of the original weight of the plastic samples [13], and multiplied by one hundred to obtain the yield in percentage [14] (Equation (1)).

(1) $$\mathrm{Yield}=\frac{\mathrm{Mass}\;\mathrm{of}\;\mathrm{obtained}\;\mathrm{fuel}}{\mathrm{Initial}\;\mathrm{weight}\;\mathrm{of}\;\mathrm{plastic}\;\mathrm{samples}}\times 100$$

Using human senses, the fuel’s color and smell were observed. With the use of an Ostwald viscometer, the fuel’s density (mass per volume) and viscosity were assessed. The fuel was added to the viscometer until it reached the volume mark, and then suction was used to draw the liquid into the top reservoir. The fuel was then allowed to flow back into the lower reservoir with the suction released, and the flow time was recorded. The viscometer’s reference material was water, and the relative viscosity of the fuel was determined using the equation below (Equation (2)):

(2) $$\eta_{rel}=\eta_{0}=\frac{\rho t}{\rho_{0}t_{0}}$$

where *ρ* is the density of the fuel, *t* is the time of fuel’s outflow, *ρ*_0_ is the density of water, *t*_0_ is the time of water’s outflow, and *η*_0_ is the viscosity of water [57]. The temperature was maintained at 60 °C throughout the process of measuring viscosity.

#### 2.3.2. Calorific Value

The calorific value is the amount of heat energy produced when a unit mass of fuel is combusted. The calorific value of the fuel was determined at the NCPC Laboratory using a Gallenkamp bomb calorimeter (Gallenkamp, Cambridge, UK), which had a scientific grade precision of 0.01 °C, using the ASTM D240-19 test method [58].

### 2.4. By-Products and Rate of Conversion of Plastic into Other Products

The by-products formed as a result of the pyrolysis reaction were collected and the rate of conversion of the plastic into other products, including final and by-products, was determined using the following formula (Equation (3)):

(3) $$\mathrm{Rate}\;\mathrm{of}\;\mathrm{conversion}\;\left(\%\right)=\frac{100-\;\mathrm{Mass}\;\mathrm{of}\;\mathrm{plastic}\;\mathrm{left}\;\mathrm{in}\;\mathrm{the}\;\mathrm{reactor}}{\mathrm{Initial}\;\mathrm{mass}\;\mathrm{of}\;\mathrm{plastic}}\times 100$$

### 2.5. Chemical Analysis

The chemical analysis of the obtained fuels was performed by Fourier transformation infrared spectroscopy (FT-IR) and by gas chromatography coupled with mass spectroscopy (GC-MS). The functional groups in each collected fuel sample were determined by FT-IR analysis using a model 8400 spectrophotometer (Shimadzu, Kyoto, Japan). Sodium chloride cells were used to perform the analysis [55] and samples were diluted in chloroform, n-hexane, and methanol. Compounds present in the obtained fuel were determined by GC-MS analysis using a QP5050A GC-MS (Shimadzu, Kyoto, Japan). Helium was used as a carrier gas and the GC column was an Elite-5ms capillary column with a maximum temperature of 350 °C and minimum bleed at 300 °C (column length: 30 cm, column diameter: 0.25 mm, and film thickness: 0.25 μm). The temperature ramping rate was 10 °C per minute up to 325 °C, after which the temperature was held for 15 min at 325 °C. The MS scan time was 1 to 45 min [59].

## 3. Results and Discussion

### 3.1. Fuel Obtained by Pyrolysis and Steel Reactor

As determined by a prior pilot lab study, the current pyrolysis method produced similar yields with or without the use of a catalyst (Ca(OH)_2_), although the non-catalyzed process required moderately longer pyrolysis times. Therefore, to keep the process cost-effective so that it could be replicated at a small scale and to eliminate pungent odors, use of the catalyst was abandoned. Another drawback of using the catalyst was the development of coke on the surface of the catalyst, which decreased the efficiency of the reaction and made recovering the catalyst from the residue difficult [60]. Moreover, without using the catalyst, water was eliminated from the product. The final product was then analyzed for physical and chemical properties.

The stainless-steel reactor designed for the pyrolysis of plastic waste proved to be efficient as it pyrolyzed most of the plastic into fuel. A key parameter of the fuel conversion process was effective cooling of the hydrocarbon vapor to avoid material loss by using ice and adequate temperature maintenance of the reactor. To maintain an appropriate temperature to ensure maximum yield, the pressure was kept constant at the start and released once the temperature crossed the required limit. Moreover, constant observations were performed to adjust to adequate parameters. Irrespective of the color, the same quality of fuel was obtained; however, differences between the physical and chemical properties were observed depending upon the type of the plastic.

### 3.2. Analysis of the Products Obtained by Pyrolysis of Plastics

Yields of the fuels obtained from the pyrolysis of five diverse types of plastics were determined. A temperature of 400–600 °C has been observed to be the most effective to produce the maximum percentage yield [32] The temperatures used in this study were comparable to the temperature of 500–550 °C determined in the study by Khan et al. [51] and Bridgwater [61] and the temperature of 400–550 °C determined by Kumar and Singh [55]. Different yields were observed depending upon the type of the plastic used in the reactor. HDPE manifested the highest percent yield of 82%, followed by PP with a yield of 61%. LDPE exhibited a yield of 58%, PS a yield of 50%, and the lowest, PET, with a yield of approximately 10%. These yields were comparable to those reported in the scientific literature. Sogancioglu et al. [45,62] also reported the highest yield from HDPE (83%), followed by PP (78%), LDPE (60%), PS (54%), and lowest from PET, whereas some studies reported a higher yield from PET [43,63] and higher liquid yield for PS due to increased reaction times. The physical characteristics of the fuel products were determined (Table 2). The fuels had distinct colors due to the colorants and additional elements used in the different plastics.

One of the parameters that determines the quality of fuel is its density. The fuel derived from PET, HDPE, PP, and PS manifested densities of 1, 0.78, 0.75, and 0.91 g/mL, respectively, while the lowest density of 0.70 g/mL was obtained for the LDPE-derived fuel. These values are significant as they are comparable to the density of commercial fuels, such as gasoline and diesel (Table 2). In a study by Khan et al. [51], the density of waste plastic pyrolysis oil was observed at 0.75 g/mL, which was comparable to the density of diesel and gasoline. Sarker et al. [64] reported a similar density for PET-derived fuel at 0.9 g/mL, and densities of 0.75, 0.76, 0.91, and 0.74 g/mL for LDPE-, HDPE-, PS-, and PP-derived fuel, respectively, were reported by Heydariaraghi et al. [65], which were all comparable to our results. While studying the characteristics of pyrolysis fuel derived from plastic waste and their mixture, Quesada et al. [42] also recorded similar results.

The viscosity of the fuel is dependent upon multiple factors, such as the feedstock, temperature range, and pyrolysis conditions, among others. Fuel consumption is directly related to the viscosity, meaning higher fuel consumption is observed with higher viscosity. Furthermore, high viscosity impacts the engine load and friction. The viscosity values of the obtained fuels are listed in Table 2. The viscosities of the fuels obtained from the pyrolysis of different plastics were in the range of 0.9 to 2.2 cSt, which were comparable to the values of high-speed diesel used commercially [51]. The viscosity of the HDPE-derived fuel was comparable to 1.63 cSt reported by Kumar and Singh [55], the viscosity of the LDPE-derived fuel was comparable to 1.27 reported by Shah et al. [66], and similarly, the viscosity of the PS-derived fuel was comparable to 1.1 cSt reported by Jahirul et al. [32]. The PP-derived fuel had a viscosity similar to 1.16 reported in the same study [32], but lower than 1.4 cSt reported by Anuar Sharuddin et al. [41]. The values were comparable to commercial fuels, which reflected their suitability for use in combustion engines.

The calorific values are expressed in MJ/kg in Table 2. The calorific values of fuels obtained from the pyrolysis of various plastics as starting materials exhibited similarity. The LDPE- and PET-derived fuels showed the highest and lowest calorific values (46.2 MJ/kg and 42.8 MJ/kg, respectively), with an absolute difference of only 3.4 MJ/kg. The average calorific value was 44.7 ± 1.9 MJ/kg, representing only a 4.3% relative standard deviation, and this average was comparable to the values of gasoline or diesel (Table 2). The calorific value of the PET-derived fuel was higher than the value reported by Anuar Sharuddin et al. [41], whereas the HDPE-derived fuel showed an approximately equal value as 45.8 MJ/kg evaluated by Kumar and Singh [55] and 46.5 MJ/kg evaluated by Singh et al. [67]. The LDPE-derived fuel also exhibited a calorific value comparable with the study Singh et al. [67]. The PS-derived fuel showed a higher calorific value than 38.4 MJ/kg reported by Heydariaraghi et al. [65], but was near the value of 40.3 MJ/kg reported by Singh et al. [67]. The high calorific values obtained from the fuel products indicated their energy potential and suitability to be used as alternative energy sources. The efficiency of fuel is also based upon this property [51]. Thus, followed by the processes of refining and distillation, the fuel obtained from the pyrolysis of plastics may be used interchangeably with fuels such as diesel or gasoline, as reported by Jahirul et al. [32].

A high conversion rate of different plastics into fuel was obtained in the study. With the exception of PET, where only a very low PTF mass yield was achieved, the calorific values corresponded to the energy conversion rates ranging from 28.6 MJ/kg plastic (PS) to 37.7 MJ/kg plastic (HDPE), determined predominantly by the PTF mass conversion yield since the calorific values of fuels obtained from different plastics were not significantly different from each other. Thus, the average energy conversion rate (with PET not considered due to the outstandingly low yield obtained) was 28.6 ± 6.8 MJ/kg plastic, representing a 23.7% relative standard deviation. In parallel to occasional low PTF mass conversion yields, some non-volatile pyrolysis residues were also observed at the end of the reaction, which were unable to be converted into fuel. These residues appeared to be waxy substances; however, they were not sufficiently significant to hinder the results of the experiments. The conversion rate of PET into fuel was determined to be 96.5%. Meanwhile, the conversion rate of HDPE into fuel was the lowest, at 93.5% with a 65 g plastic residue. The highest conversion rate of 99% was observed for PP and no significant residue was observed in the reactor after the pyrolysis. High conversion rates point to the ability of the plastics to be successfully converted into fuel alternative products using a cost-effective method.

### 3.3. Chemical Analysis of Fuel by FT-IR

FT-IR of the pyrolysis-derived fuels presented notable results in the study (Table 3, also see Supplementary Materials Figure S5). The PET-derived fuel exhibited the presence of functional groups such as alkene, alkane, carbonyl, and ether. A peak at 873 cm^−1^ indicated the presence =C-H groups, whereas the peak at 1425 cm^−1^ indicated the presence of –C-H groups. A peak at 1020 cm^−1^ showed the presence of C-O functional groups, which was also found by Jia et al. [63], in which the presence of C-O functional groups at 1020 and 1085 cm^−1^ confirmed the additional presence of ether functional groups. Studies by Sarker et al. [64], Rapsing [68], and Sarker and Rashid [64] also confirmed the presence of alkenes, alkanes, carbonyl, and ether functional groups. It was observed that compared to the alkene functional groups, alkanes showed stretching variations (Table 3).

The results of FT-IR of the HDPE-derived fuel indicated the presence of three significant functional groups: aldehydes, alkenes, and alkanes (Table 3). The peak at 873 cm^−1^ confirmed the presence of the alkane functional group, whereas the peak at 1411 cm^−1^ represented the alkane functional group. The spectrum obtained from FT-IR in the current study was comparable to that reported by Olufemi and Olagboye [69] for HDPE-derived fuel. For instance, the peak at 2850 cm^−1^ was comparable to the peak at 2853 cm^−1^ obtained in the study that confirmed the presence of the alkene functional group. The bending of the peak representing the –C-H bond and CH_2_ bond at 1425 cm^−1^ was comparable to the results shown by Imtiaz et al. [14]. Similarly, the results were comparable to the peaks at 2916, 2851, and 1624 cm^−1^ identified by Kumar and Singh [70] for alkane and alkene stretching, respectively, for HDPE-derived fuel. Hence, the composition of the HDPE-derived fuel indicated its applicability as a fuel since the most significant functional groups present in diesel and gasoline were also present in the HDPE-derived fuel.

FT-IR of the LDPE-derived fuel indicated the presence of alkane, alkene, and aromatic functional groups (Table 3). The peak obtained at 2920 cm^−1^ represented the presence of the alkane functional group (C-H) with stretching vibration. Similarly, the peaks at 1618, 1419, and 873 cm^−1^ showed alkenes with C=C stretching vibration, alkanes with C-H bending, and alkenes with C-H bending vibration, respectively. Since the C-C bond in LDPE is very weak, it is easily converted into double bonds of alkenes when subjected to thermal degradation. Shah et al. [66] also indicated the presence of alkanes, alkenes, and aromatic compounds in pyrolysis fuel. The peaks at 1603 and 2919 cm^−1^ corresponded to those at 1618 and 2918 cm^−1^ that were also identified as stretching vibration in alkenes and alkanes by Siddiqui and Redhwi [71]. However, the peak at 3084 cm^−1^ identified by Siddiqui and Redhwi [71] was not exhibited in the results due to the pyrolysis of separate plastics.

The results obtained by FT-IR for the PP-derived fuel exhibited two significant peaks at 873 and 1413 cm^−1^, indicating =C-H and –C-H functional groups, respectively. Alkanes and alkenes were found to be the most significant functional groups. Similarly, for the PS-derived fuel, a peak at 1541 cm^−1^ indicated the presence of aromatics with C=C stretching. Furthermore, the peaks at 1680,1396, and 752 cm^−1^ showed the presence of alkenes with C=C stretching, alkanes with C=C bending, and alkenes with =C-H bending vibrations, respectively. The presence of these functional groups and comparable peaks were also identified by Ahmad et al. [72] and Singh et al. [67], which supported these results. The aromatic compounds showed an increasing trend with temperature.

The FT-IR analysis of the PS-derived fuel exhibited peaks at wavenumbers 752, 1396, 1491, 1541, 1680, 2922, and 3024 cm^−1^. These peaks were also identified by Singh et al. [67]. They found comparable peaks at 1634, 2929, and 3027 cm^−1^, which confirmed the presence of stretching vibrations in alkenes and alkanes. The FT-IR spectra of all five fuel types revealed a similar functional group composition. The most common functional groups were =C-H (alkenes) and C-H (alkanes). All of the fuels had peaks ranging from 675 to 1000 cm^−1^, indicating the existence of alkenes (=C-H). Similarly, the existence of a band from 1400 to 1600 cm^−1^ verified the presence of C=C, implying the presence of olefins. The presence of a C-H bond was confirmed by the appearance of peaks ranging from 2850 to 3000 cm^−1^. This also revealed that CH_2_ and CH_3_ had been stretched into C-H. Overall, the bending vibrations outnumbered the stretching vibrations. A fuel burns more effectively if there are more alkanes present. Fuel extracted from PET was the only fuel that demonstrated the presence of ether, with the FT-IR spectrum demonstrating the existence of the C-O functional group. Ether was present because it was present in PET, which breaks down to produce alkyl aryl ether.

### 3.4. Chemical Analysis of Fuel by GC-MS

GC-MS was performed on the fuels obtained by pyrolysis and results regarding their composition were obtained (Table 4, also see Supplementary Materials Figure S6). A study by Sarker et al. [64] indicated the presence of chains of hydrocarbons lying in the range of C_6_–C_27_. For the PET-derived fuel, a hydrocarbon range of C_9_–C_24_ was observed in the study. GC-MS revealed the existence of biphenyl and quaterphenyl in the fuel derived from PET, which was further supported by the FT-IR data that indicated the presence of a carbonyl group (Table 4). Jia et al. [63] detected comparable results, but observed that carbonyl groups and aromatic ether decreased by approximately 50% after the use of zeolite as a catalyst. Anuar Sharuddin et al. [41] and Quesada et al. [42] also reported similar findings with the presence of methyl styrene, benzophenone, and benzoic compounds, among others.

The carbon numbers of the aliphatic hydrocarbons in the fuel generated from HDPE were found to be between C_10_ and C_21_. Tetradecane was among the several alkanes and alkenes that Olufemi and Olagboye [69] confirmed to be present in the fuel (Table 4). GC-MS of the HDPE-derived fuel confirmed the presence of alkanes and alkenes, as seen in the FT-IR analysis. A study by Kumar and Singh [55] also reported comparable results, indicating the presence of groups such as decene, octadecene, and other functional groups. They also reported a similar retention time at 18.14 min for heneicosane.

Numerous aliphatic hydrocarbons between C_10_ and C_28_ were detected in the LDPE-derived fuel by GC-MS. Lengthy and straight alkane and alkene chains were observed. All chemicals from this study were also included in the report by Sarker et al. [59]. The liquid fuel contained 18.1% heavy oil, 54.5% diesel fuel, and 27.2% gasoline, as reported by Sarker et al. [59]. Akgün et al. [73] and Shah et al. [66] also reported the composition of LDPE-derived fuel and the presence of decene and octadecene groups. This indicated that the fuel was comparable to diesel fuel and could be used in stoves, heavy cars, central heating systems, and railways (Table 4). the fuel could also be used in smaller combustion engines as well. In the current study, the PP-derived fuel had a methyl group present (Table 4), which was also reflected in the study by Sarker and Rashid [59]. These results were also comparable to the findings of Akgün et al. [73]. The study described hydrocarbons of various ranges that were contained in the C_9_–C_15_ range. Sarker and Rashid [59] also verified the presence of methyl and tetradecene. The majority of the compounds had a cyclohexane ring. Sarker et al. [59] also reported the presence of methylstyrene (C_9_H_10_). The PS-derived fuel exhibited the presence of methyl and benzene rings, which was comparable with past studies (Table 4). Siddiqui and Redhwi [71] and Sarker et al. [59] reported the presence of biphenyl (C_12_H_10_), bibenzyl (C_14_ H_14_), and diphenylmethane (C_13_ H_12_). Jung et al. [74] reported comparable findings with the presence of styrene and benzene compounds, which was further supported by the results from Siddiqui [75] reporting the presence of phenyl and bi-phenyl compounds. Due to the styrene content in the PS-derived fuel, it could be used in chemical synthesis applications and also be utilized in a mixture. The results of GC-MS showed that the compounds present in the pyrolysis fuels were also found in commercial fuels, such as diesel. These compounds included, among other groups, benzene methyl styrene, naphthalene, and octadecene, which are found in both diesel and pyrolysis fuel products [76].

The results of this study showed that the fuel products from pyrolysis of five different types of plastics could be successfully achieved in a cost-effective and sustainable manner, considering the conditions of developing countries such as Pakistan. The properties of the fuels were comparable to conventional non-renewable diesel and gasoline fuels. However, because of their high calorific values, they can also be used as an ignition source in domestic houses for heating, cooking, burning in boilers, etc. With further refining and distillation, the fuels could be used in combustion engines. One study found that the high brake thermal efficiency attained from a blend of pyrolysis fuels was comparable to the threshold for diesel engines, but the fuel emitted more gases [29]. This is because the fuel needed further refinement and the unsaturated hydrocarbons present in the pyrolysis oils hindered the process to achieve similar results as diesel in combustion engines [71]. While reviewing the use of pyrolysis oil as a substitute for transport fuel, Jahirul et al. [32] concluded that although further processing of pyrolysis fuel was required to attain results similar to the standards in diesel combustion engines, the fuel could be used directly for ignition as furnace oil in industries or in domestic circles. Cost-effectiveness is significant to promote this technology at smaller and larger scales. A positive return on investment (ROI) can be attained if greater volumes of plastics are pyrolyzed or the process is made economically feasible [32]. This study therefore indicates that pyrolysis fuels obtained from a simple and cost-effective process can be used as fuel alternatives at small and large scales. The method is easy to replicate even at domestic levels, and the use of these fuels for ignition will significantly reduce the dependency on conventional non-renewable fuels and mitigate the major problems of the energy crisis [77] and plastic waste at the same time [10]. Further studies, such as thermogravimetric analysis, can be performed in the future to assess the thermal characteristics of the fuels and weight changes. Furthermore, collaboration with refineries through government and private initiatives is required to promote large-scale research in the use of pyrolysis oils in internal combustion engines. Furthermore, at a smaller scale, due to the simplicity and effectiveness of the method, it can be replicated and encouraged to reduce the reliance on non-renewable fuels.

## 4. Conclusions

The study aimed to contribute towards solving two key issues of developing countries such as Pakistan: the energy crisis and plastic waste management. The results indicate that compared to conventional fuels, the pyrolysis of five different plastics can generate products that can be utilized to meet the energy needs and minimize plastic waste. Pyrolysis fuel attained from HDPE showed the highest yield of 82% whereas PET provided the lowest yield of 10.7%. The yields from LDPE, PP, and PS were found to be 58.0%, 61.8%, and 50.0% respectively. All of the products showed a high calorific value, with the LDPE-derived fuel having the highest value of 46.21 MJ/kg, followed by PP with 46.19 MJ/kg, HDPE with 45.88 MJ/kg, and PS with 45.80 MJ/kg. These values were comparable to the calorific values of diesel (45.50 MJ/kg) and gasoline (45.80 MJ/kg). The viscosities of the pyrolysis fuels were also similar to those of conventional fuels. FT-IR and GC-MS analysis of the pyrolysis products indicated the presence of compounds similar to those in gasoline and diesel; however, the presence of unsaturated compounds may hinder their efficiency in combustion engines. Therefore, through processes such as hydrogenation, the quality of the fuel can be further improved to meet the standards of diesel and gasoline in internal combustion engines. Due to the high calorific values, the fuels can be used as energy resources in the form of furnace oil, in boilers, or for domestic use in cooking or heating. Improvements are required in the future to make the process more efficient and refine the products. The residual products can also be reused to produce other products. Improvements in the design of the reactor, such as installing a more efficient condenser or installing two condensers, would minimize the loss of hydrocarbons to increase the product yield. The fuel needs to be fractionally distilled and refined before use to give higher efficiency in engines during combustion. The research has also demonstrated its success by producing effective findings in terms of the fuel’s quality and quantity using a cost-effective and simple method. The quality and viability of the process for use in the country by waste management authorities might be improved with further study on the produced hydrocarbons and the reactor design. Promotion of the technique through private and government initiatives and research to refine the quality of the products is required in the future. This can also increase entrepreneurship opportunities at small and large scales in developing countries such as Pakistan and simultaneously solve the problems of waste management and the energy crisis, while contributing to the circular economy model.

## Figures and Tables

**Table 1:** Plastic types used in the study and their typical technical applications.

Name	Abbreviation	Symbol	Application
Polyethylene terephthalate	PET	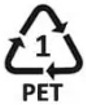	Water and soft drink bottles, cooking oil containers, etc.
High-density polyethylene	HDPE	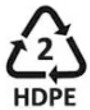	Bottle caps, shampoo, and detergent containers, etc.
Polyvinyl chloride	PVC	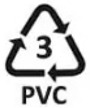	Pipes, food foils, etc.
Low-density polyethylene	LDPE	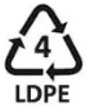	Shopping bags, wrappings, etc.
Polypropylene	PP	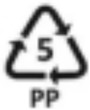	Disposable utensils, furniture, toys, etc.
Polystyrene	PS	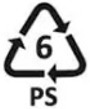	Refrigerator trays, CD cases, disposable cups/plates, etc.
Others	–	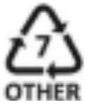	Makeup/cream bottles (polycarbonates), etc.

**Table 2:** Conversion yields and physical parameters of the fuels obtained from different plastic types.

Plastic Type ^1^	Fuel				Energy
	Yield (%)	Density (g/mL)	Viscosity ^2^ (cSt)	Calorific Value (MJ/kg Fuel)	Conversion (MJ/kg Plastic)
PET	10.7	1.00	2.20	42.4	4.5
HDPE	82.2	0.78	1.60	45.9	37.7
LDPE	58.0	0.70	1.41	46.2	26.8
PP	61.8	0.75	1.58	46.2	28.6
PS	50.0	0.91	0.99	42.8	21.4
Gasoline	–	0.74	1.70 ^3^	45.8	–
Diesel	–	0.83	2.61 ^3^	45.5	–

^1^ Abbreviations: PET—polyethylene terephthalate; HDPE—high density polyethylene; LDPE—low density polyethylene; PP—polypropylene; PS—polystyrene. ^2^ Viscosity determined at 60 °C. ^3^ Viscosity determined at 40 °C.

**Table 3:** FT-IR assignment for the derived fuel products.

Sr. No.	Wave Number (cm^−1^)	Functional Group	Type of Variation	Nature of Functional Group
PET				
1.	698	=C-H	Bending	Alkene
2.	798	=C-H	Bending	Alkene
3.	873	=C-H	Bending	Alkene
4.	1425	-C-H	Bending	Alkane
5.	1020	C-O	Stretching	Alkyl Aryl Ether
6.	1618	C=C	Stretching	Alkene
7.	2918	=C-H	Stretching	Alkane
HDPE				
1.	798	=C-H	Bending	Alkene
2.	873	=C-H	Bending	Alkene
3.	1411	-C-H	Bending	Alkane
4.	1624	C=C	Stretching	Alkene
5.	2850	=C-H	Stretching	Alkene
6.	2916	C-H	Stretching	Alkane
LDPE				
1.	873	C-H	Bending	Alkenes
2.	1016	C-H	Bending (in plane)	Aromatic hydrocarbons
3.	1419	C-H	Bending	Alkanes
4.	1618	C=C	Stretching	Alkenes
5.	2920	C-H	Stretching	Alkanes
PP				
1.	711	=C-H	Bending	Alkene
2.	798	=C-H	Bending	Alkene
3.	873	=C-H	Bending	Alkene
4.	1413	-C-H	Bending	Alkane
5.	2918	C-H	Stretch	Alkane
PS				
1.	752	=C-H	Bending	Alkenes
2.	1396	C=C	Bending	Alkanes
3.	1491	C-C	Stretching (in ring)	Aromatics
4.	1541	C=C	Stretching	Aromatics
5.	1680	C=C	Stretching	Alkenes
6.	2922	C-H	Stretching	Alkanes
7.	3024	=C-H	Stretching	Alkenes

**Table 4:** GC-MS assignment for the derived fuel products.

Sr. No.	Retention Time (min)	Compound	Molecular Formula	Molecular Weight
PET				
1	3.36	α-Methyl Styrene	C_9_H_10_	118
2	4.40	Benzaldehyde dimethyl acetal	C_9_H_12_O_2_	152
3	5.92	Benzoic acid	C_7_H_6_O_2_	122
4	9.24	Biphenyl	C_12_H_10_	154
5	13.32	Benzophenone	C_13_H_10_O	182
6	22.62	1,1′:3′,1′:3′,1′′′-Quaterphenyl	C_24_H_18_	306
HDPE				
1	3.32	1-Decene	C_10_H_20_	140
2	4.32	3-Tetradecene	C_14_H_28_	196
3	5.79	3-Tetadecene	C_14_H_28_	196
4	7.35	Tridecane	C_13_H_28_	184
5	9.28	Tetradecane	C_14_H_30_	198
6	10.59	Octadecane	C_18_H_38_	254
7	12.15	Hexadecane	C_16_H_34_	226
8	13.69	Nonadecane	C_19_H_40_	268
9	15.06	Heneicosane	C_21_H_44_	296
10	16.44	Heneicosane	C_21_H_44_	296
11	17.74	Octacosane	C_28_H_58_	394
12	18.95	Heneicosane	C_21_H_44_	296
LDPE				
1	3.27	1-Decene	C_10_H_20_	140
2	4.39	1-Undecene	C_11_H_22_	154
3	5.81	1-Dodecene	C_12_H_24_	168
4	7.38	1-Tridecene	C_13_H_26_	182
5	9.02	1-Pentadecene	C_15_H_30_	210
6	12.14	Heptadecene	C_17_H_36_	238
7	13.70	Octadecane	C_18_H_38_	254
8	15.11	Nonadecane	C_19_H_40_	268
9	16.45	Eicosane	C_20_H_42_	282
10	17.73	Heneicosane	C_21_H_44_	296
11	20.13	Octacosane	C_28_H_58_	394
PP				
1	3.39	α-Methylstyrene	C_9_H_10_	118
2	4.34	3-Tetradecene	C_14_H_28_	196
3	4.97	3-Octadecene	C_18_H_36_	252
4	7.67	3-Octadecene	C_18_H_36_	252
5	8.40	3-Octadecene	C_18_H_36_	252
6	11.15	Nonadecane	C_19_H_40_	268
7	13.54	Benzene, 1,1′-(1,3-propanediyl)bis-	C_15_H_16_	196
PS				
1	3.41	α-Methylstyrene	C_9_H_10_	118
2	3.87	Benzene, 2-propenyl	C_9_H_10_	118
3	9.30	Biphenyl	C_12_H_10_	154
4	10.14	Diphenylmethan	C_13_H_12_	168
5	11.55	Bibenzyl	C_14_H_14_	182
6	13.58	Benzene, 1, 1′-(1,3propanediyl) bis-	C_15_H_16_	196
7	14.65	Benzene, 3-butenyl	C_10_H_12_	132
8	18.22	Naphthalene, 2-phenyl-	C_16_H_12_	204
9	19.85	Naphthalene, 2-phenlymethyl	C_17_H_14_	218

## Data Availability

Not applicable.

## Supplementary Materials

The following supporting information can be downloaded at: https://www.mdpi.com/article/10.3390/polym15010024/s1, Figure S1: Feedstocks from dumpsites in the study; Figure S2: Preliminary pilot study set-up; Figure S3: Pyrolysis fuel emerging from the reactor; Figure S4: Pyrolysis fuels produced from the reactor; Figure S5: FT-IR spectra of plastic-derived fuels by plastic type. (**a**) PET-derived fuel; (**b**) HDPE-derived fuel; (**c**) LDPE-derived fuel; (**d**) PP-derived fuel; (**e**) PS-derived fuel; Figure S6: GC-MS spectra of plastic-derived fuels by plastic type. (**a**) PET-derived fuel; (**b**) HDPE-derived fuel; (**c**) LDPE-derived fuel; (**d**) PP-derived fuel; (**e**) PS-derived fuel.
