# Supplementary material for: Analysis of Fuel Alternative Products Obtained by the Pyrolysis of Diverse Types of Plastic Materials Isolated from a Dumpsite Origin in Pakistan
Source: Polymers (Basel). 2022 Dec 21;15(1):24. doi: 10.3390/polym15010024 (PMC9823855; doi:10.3390/polym15010024)
Supplement: Supplementary file 1 [file polymers-15-00024-s001.zip › polymers-2052689-supplementary.pdf]

# Analysis of fuel alternative products obtained by the pyrolysis of diverse types of plastic materials isolated from a dumpsite origin in Pakistan

Nuzhat Javed [1], Sana Muhammad [1], Shazia Iram [1,*], Muhammad Wajahat Ramay [1,2], Shaan Bibi Jaffri [1], Mariem Damak [2], György Fekete [2], Zsolt Varga [2], András Székács [2] and László Aleksza [2,3,*]

[1] Department of Environmental Sciences, Fatima Jinnah Women University, The Mall, Rawalpindi, 46000, Pakistan
[2] Institute of Environmental Sciences, Hungarian University of Agriculture and Life Sciences, Páter Károly u. 1, H-2100 Gödöllő, Hungary
[3] Profikomp Environmental Technologies Inc., Kühne Ede u. 7, H-2100 Gödöllő, Hungary
* Correspondence: shaizairam@fjwu.edu.pk (S.I.); aleksza.laszlo@uni-mate.hu (L.A.); Tel.: +92-51-9292900-(2083) (S.I.); +36-28-512-490 (L.A.)

## Supplementary Materials

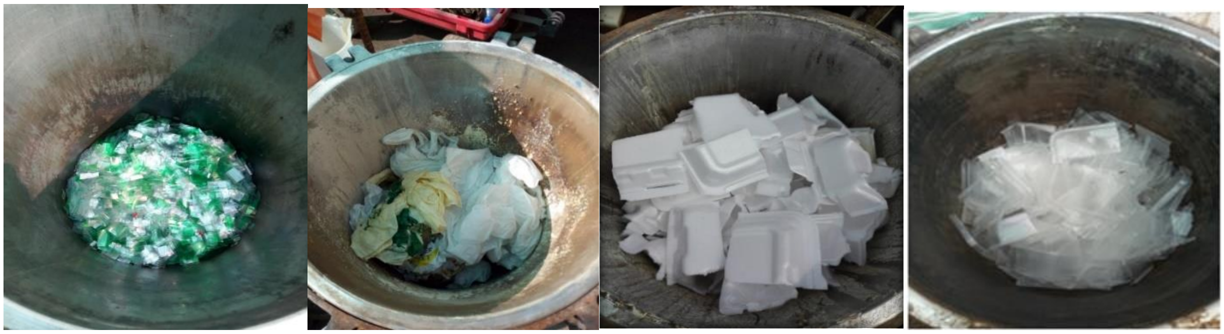

**Figure S1.** Feedstocks from dumpsites in the study.

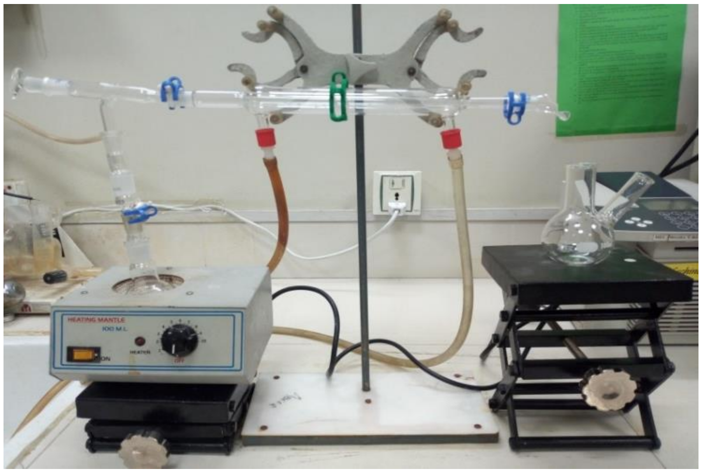

**Figure S2.** Preliminary pilot study setup.

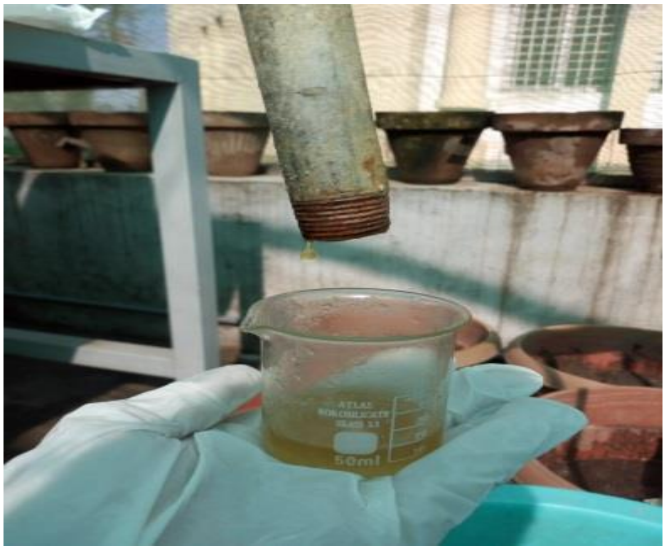

**Figure S3.** Pyrolysis fuel emerging from the reactor.

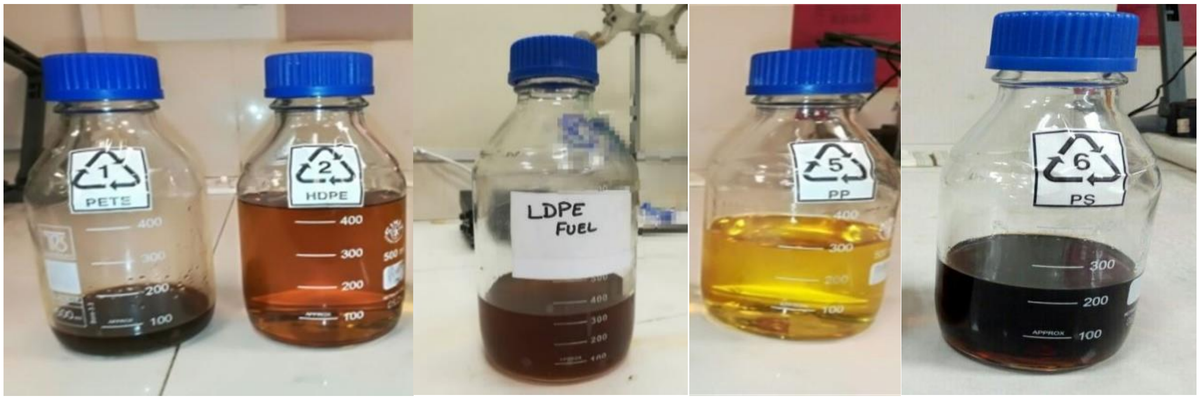


**Figure S4.** Pyrolysis fuels produced from the reactor.

(a)

(b)

(c)

(d)

(e)

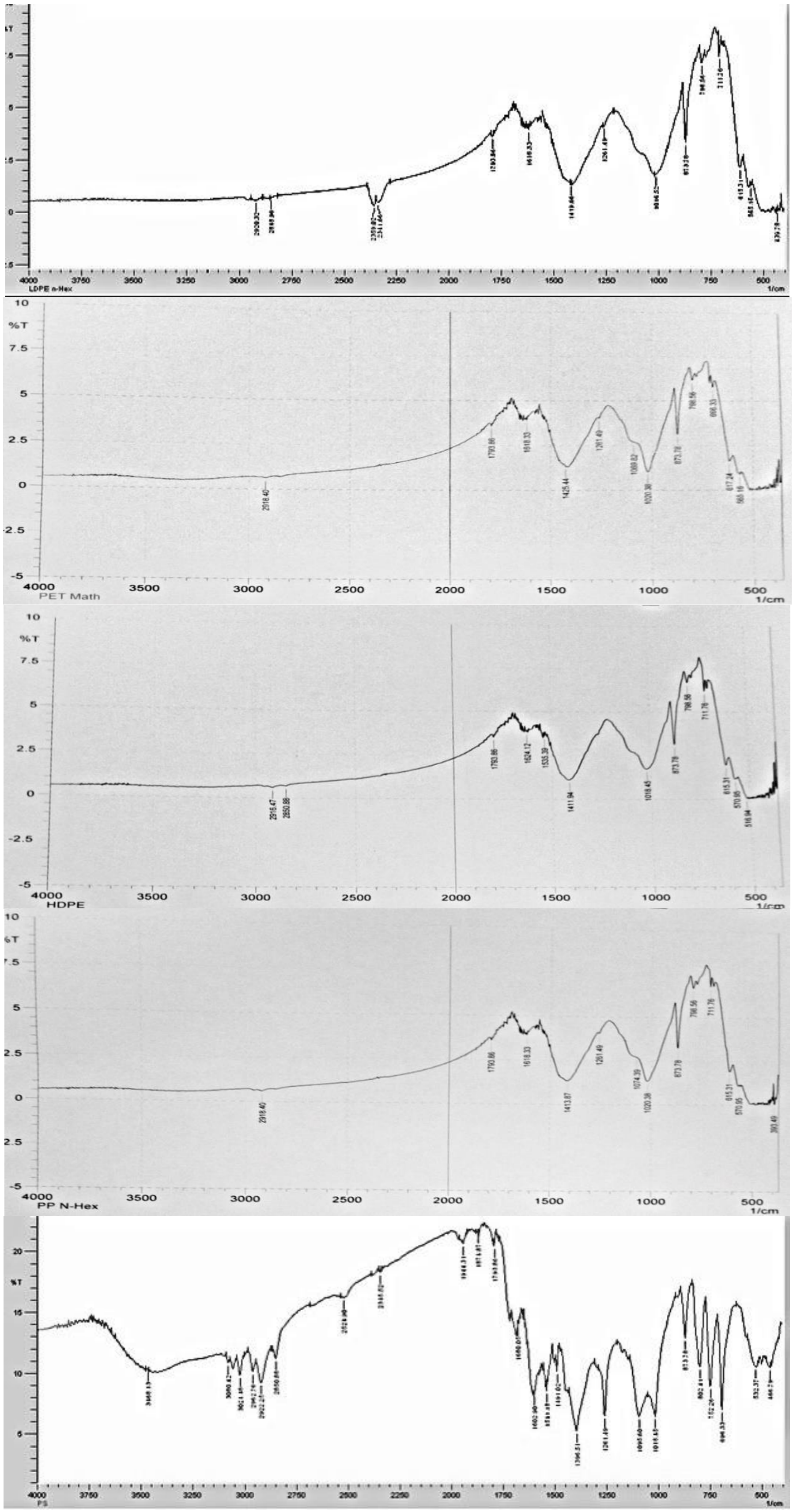


**Figure S5.** FT-IR spectra of plastic-derived fuels by plastic type. **(a)** PET-derived fuel; **(b)** HDPE-derived fuel; (c) LDPE-derived fuel; (d) PP-derived fuel; (e) PS-derived fuel.

(a)

(b)

(c)

(d)

(e)

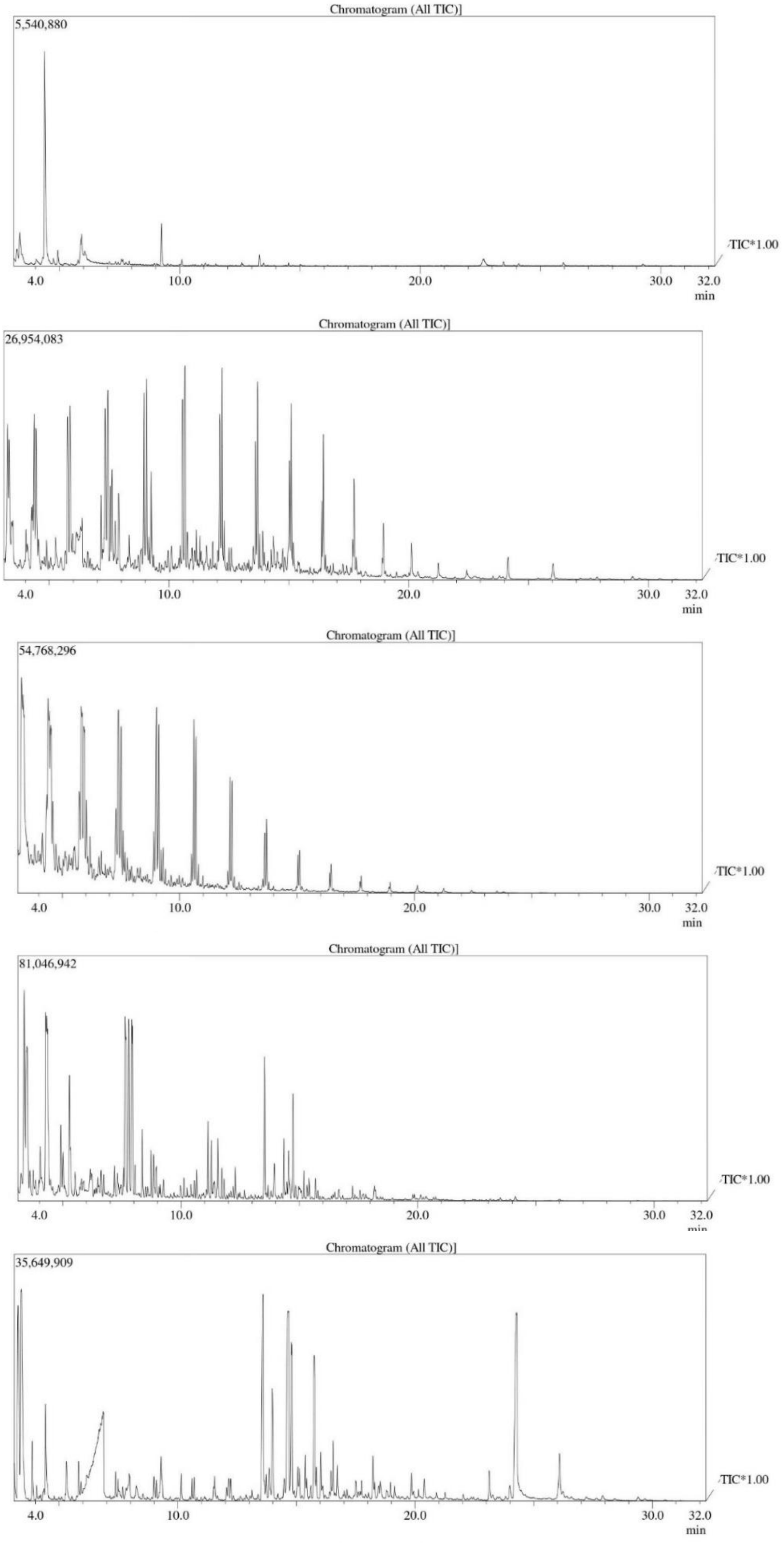


**Figure S6.** GC-MS spectra of plastic-derived fuels by plastic type. **(a)** PET-derived fuel; **(b)** HDPE-derived fuel; (c) LDPE-derived fuel; (d) PP-derived fuel; (e) PS-derived fuel.
